# Incidence of New Onset Asthma after the World Trade Center Disaster

**DOI:** 10.1289/ehp.11331

**Published:** 2008-07

**Authors:** Albert Miller

**Affiliations:** Pulmonary Medicine, Caritas Health Care, Mary Immaculate Hospital, Jamaica, New York, E-mail: almiller@bqhcny.org

Wheeler et al. (2007) provided useful information on the well-recognized problem of airways disease resulting from World Trade Center (WTC) exposure, reporting a 3-year risk of 3.6% for new physician-diagnosed asthma. As a pulmonologist in New York City who has also treated many WTC workers in a dedicated program, I would like to share the perspectives that my clinician colleagues have shared with me.

The diagnosis of asthma, even if made by physicians, is often nonspecific and based on symptoms that are nonspecific, as well as common. Diagnostic clues such as chronicity, recurrence, response to therapy, and variability in pulmonary function are not available on the first visit or the first few visits. A diagnosis bias toward asthma may operate for many reasons: *a*) a group under surveillance has an increased awareness of the target disease; *b*) asthma has been widely publicized to physicians, as well as to the public, as a result of WTC exposure; *c*) lists of accepted diagnoses required on first visits by monitoring and treatment programs, insurance companies, and compensation systems may guide the physician’s diagnosis to asthma even if he/she is not certain that this diagnosis has been established; and *d*) bias may exist in specifying the start of an ongoing illness, so that patients tend to associate it with a remarkable event like the WTC disaster even if symptoms or a physician’s diagnosis preceded this event. Wheeler et al. (2007) recognized the difficulty of estimating the incidence of disease, given the propensity of patients to cite a diagnosis that may not have been substantiated and to present for care only if symptomatic.

Wheeler et al. (2007) noted that even if all exposed persons were included in the denominator, the incidence of new asthma was still high. Much weight is placed on the estimated incidence of new asthma in the general population, for which the authors cited a review article, which in turn, cited a study from rural Minnesota that ended 25 years ago (Yungingen et al. 1992). Incidence of asthma is affected by region (including rural vs. inner city), occupation, smoking, temporality, and other factors.

Wheeler et al. (2007) have brought their information to public attention to be confirmed by more specific criteria for diagnosis, longer clinical follow-up, and additional estimates of incidence in relevant urban populations.

## Editor’s note

In accordance with journal policy, Wheeler et al. were asked whether they wanted to respond to this letter, but they chose not to do so.

## References

[b1-ehp0116-a0286a] Wheeler K, McKelvey W, Thorpe L, Perrin M, Cone J, Kass D (2007). Asthma diagnosed after 11 September 2001 among rescue and recovery workers: findings from the World Trade Center Health Registry. Environ Health Perspect.

[b2-ehp0116-a0286a] Yungingen JW, Reed CE, O’Connell J, O’Fallon WM, Silverstein MD (1992). A community-based study of the epidemiology of asthma. Incidence rates, 1964–1983. Am Rev Respir Disease.

